# Supplementary material for: Influences of chemotype and parental genotype on metabolic fingerprints of tansy plants uncovered by predictive metabolomics
Source: Sci Rep. 2023 Jul 19;13:11645. doi: 10.1038/s41598-023-38790-7 (PMC10356770; doi:10.1038/s41598-023-38790-7)
Supplement: Supplementary file 1 — Supplementary Figures. [file 41598_2023_38790_MOESM1_ESM.pptx]

## Slide 1
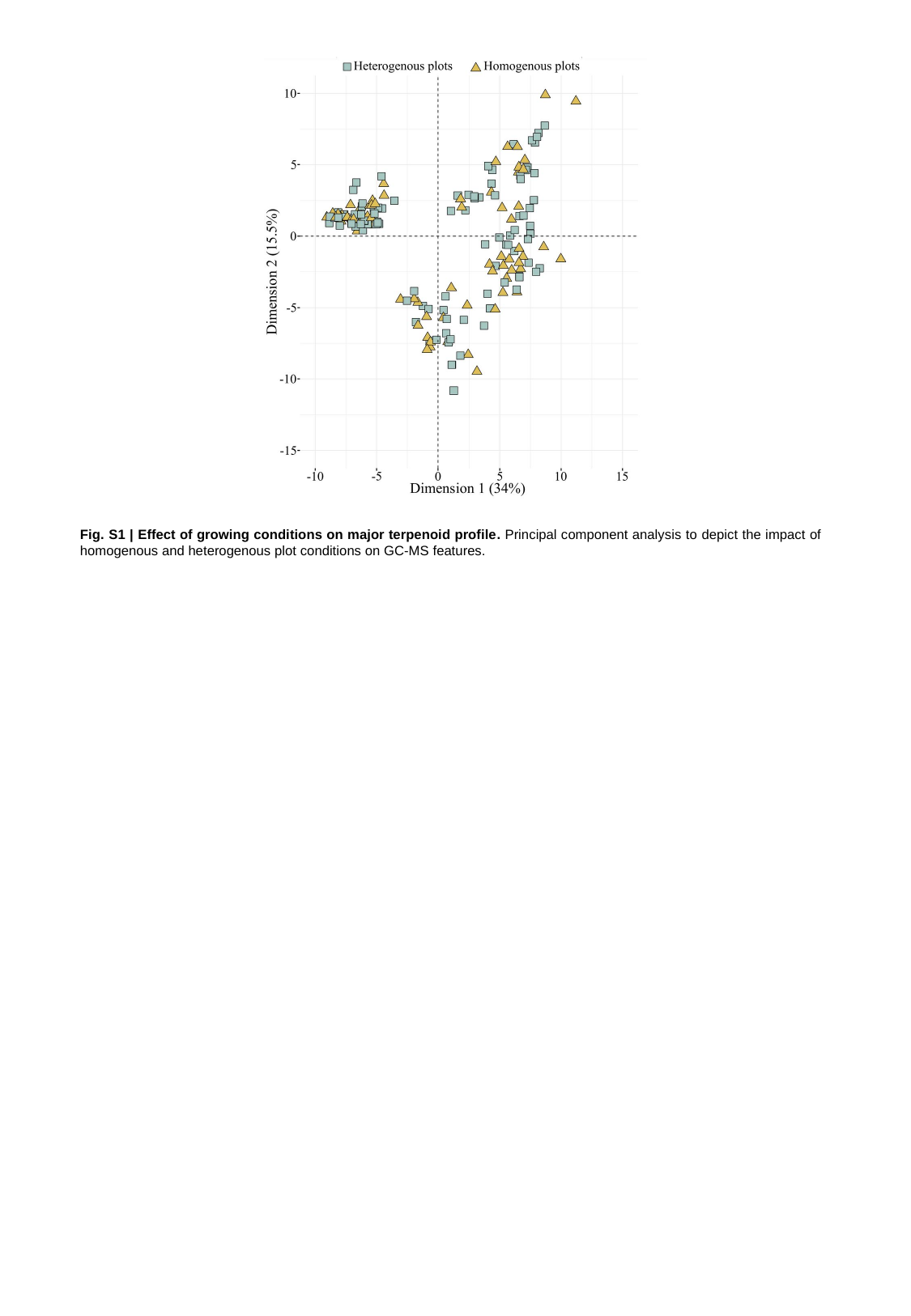

Fig. S1 | Effect of growing conditions on major terpenoid profile. Principal component analysis to depict the impact of homogenous and heterogenous plot conditions on GC-MS features.

## Slide 2
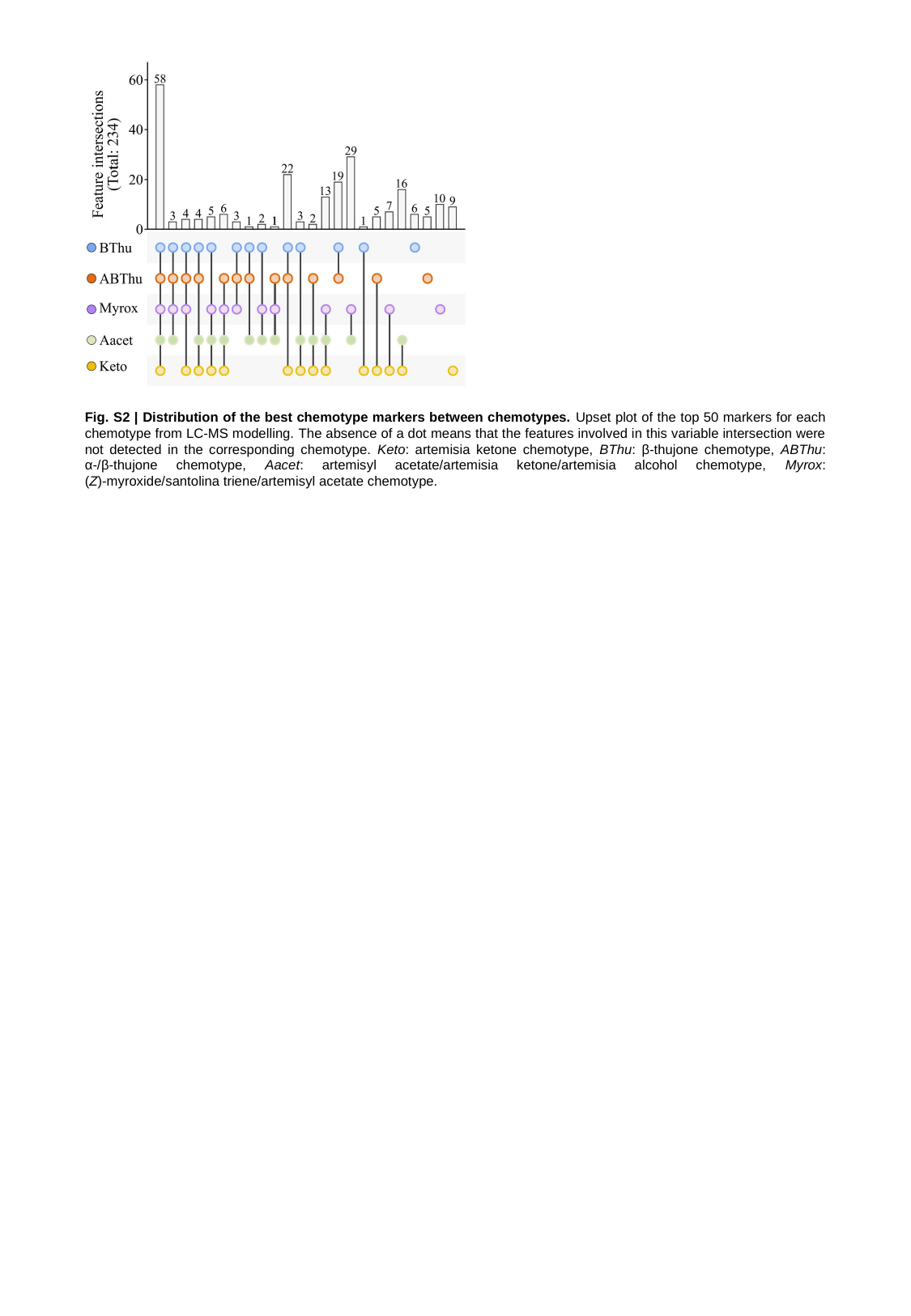

Fig. S2 | Distribution of the best chemotype markers between chemotypes. Upset plot of the top 50 markers for each chemotype from LC-MS modelling. The absence of a dot means that the features involved in this variable intersection were not detected in the corresponding chemotype. Keto: artemisia ketone chemotype, BThu: β-thujone chemotype, ABThu: α-/β-thujone chemotype, Aacet: artemisyl acetate/artemisia ketone/artemisia alcohol chemotype, Myrox: (Z)-myroxide/santolina triene/artemisyl acetate chemotype.

## Slide 3
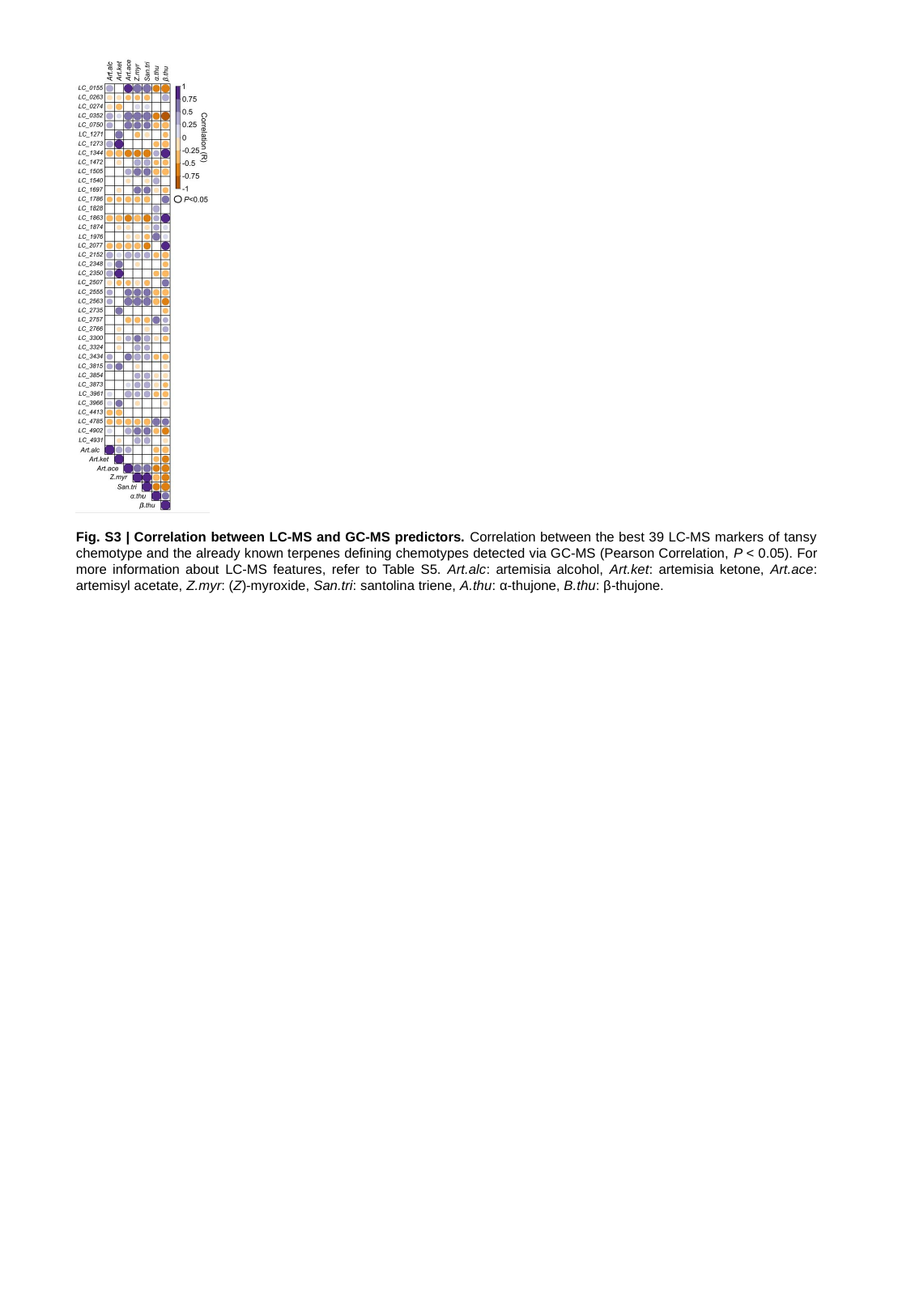

Fig. S3 | Correlation between LC-MS and GC-MS predictors. Correlation between the best 39 LC-MS markers of tansy chemotype and the already known terpenes defining chemotypes detected via GC-MS (Pearson Correlation, P < 0.05). For more information about LC-MS features, refer to Table S5. Art.alc: artemisia alcohol, Art.ket: artemisia ketone, Art.ace: artemisyl acetate, Z.myr: (Z)-myroxide, San.tri: santolina triene, A.thu: α-thujone, B.thu: β-thujone.

## Slide 4
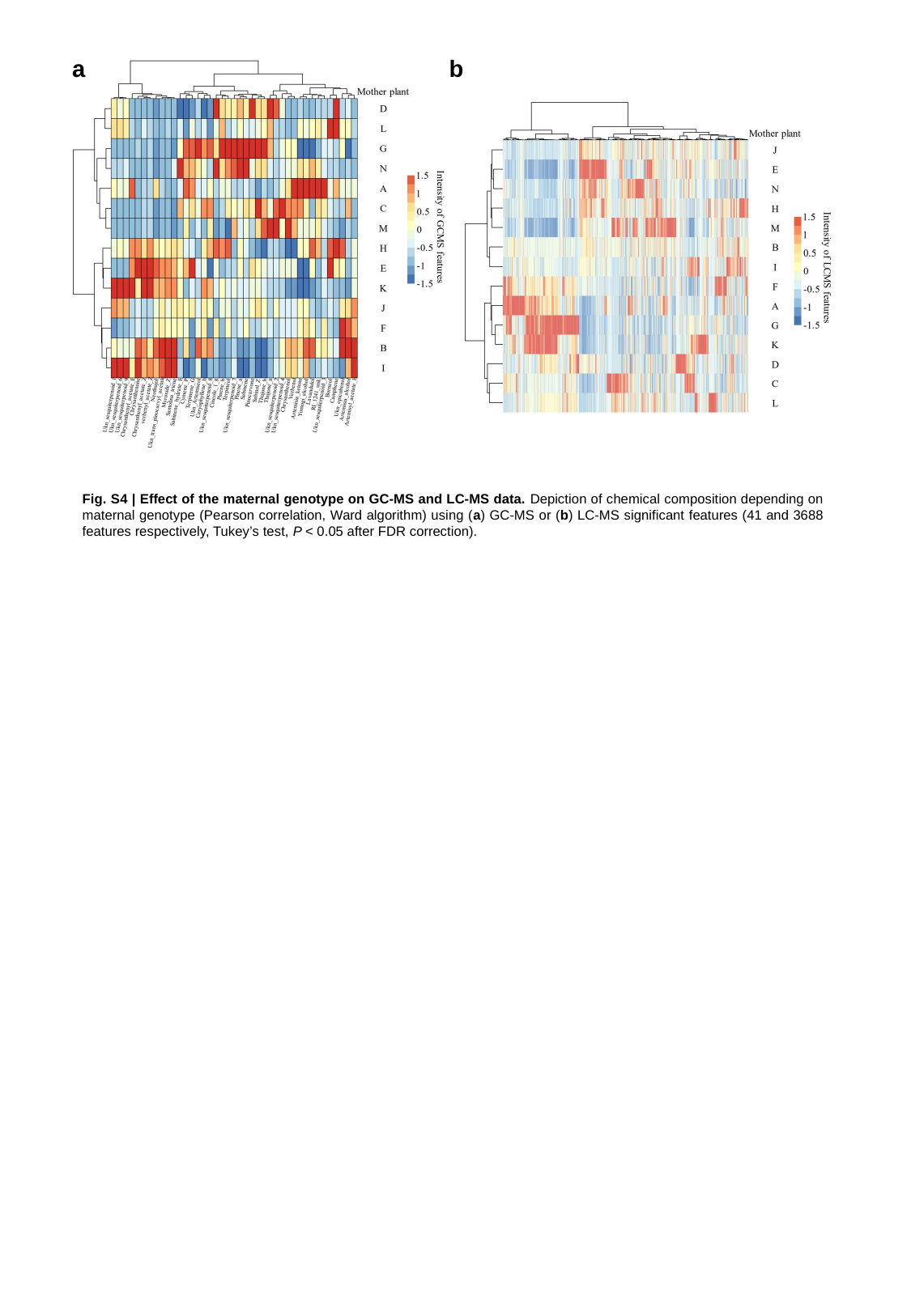

a
b
Fig. S4 | Effect of the maternal genotype on GC-MS and LC-MS data. Depiction of chemical composition depending on maternal genotype (Pearson correlation, Ward algorithm) using (a) GC-MS or (b) LC-MS significant features (41 and 3688 features respectively, Tukey’s test, P < 0.05 after FDR correction).

## Slide 5
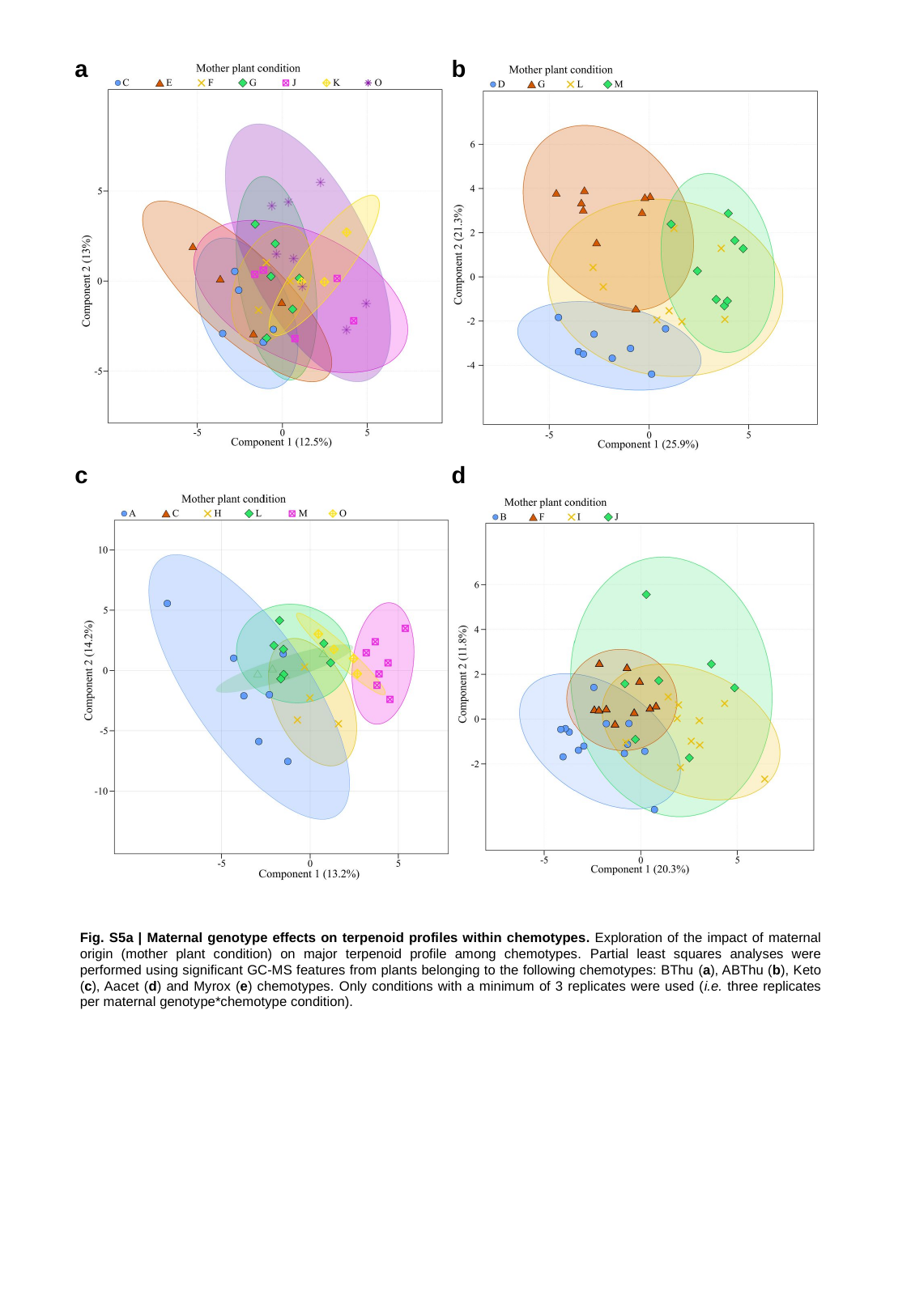

a
b
c
d
Fig. S5a | Maternal genotype effects on terpenoid profiles within chemotypes. Exploration of the impact of maternal origin (mother plant condition) on major terpenoid profile among chemotypes. Partial least squares analyses were performed using significant GC-MS features from plants belonging to the following chemotypes: BThu (a), ABThu (b), Keto (c), Aacet (d) and Myrox (e) chemotypes. Only conditions with a minimum of 3 replicates were used (i.e. three replicates per maternal genotype*chemotype condition).

## Slide 6
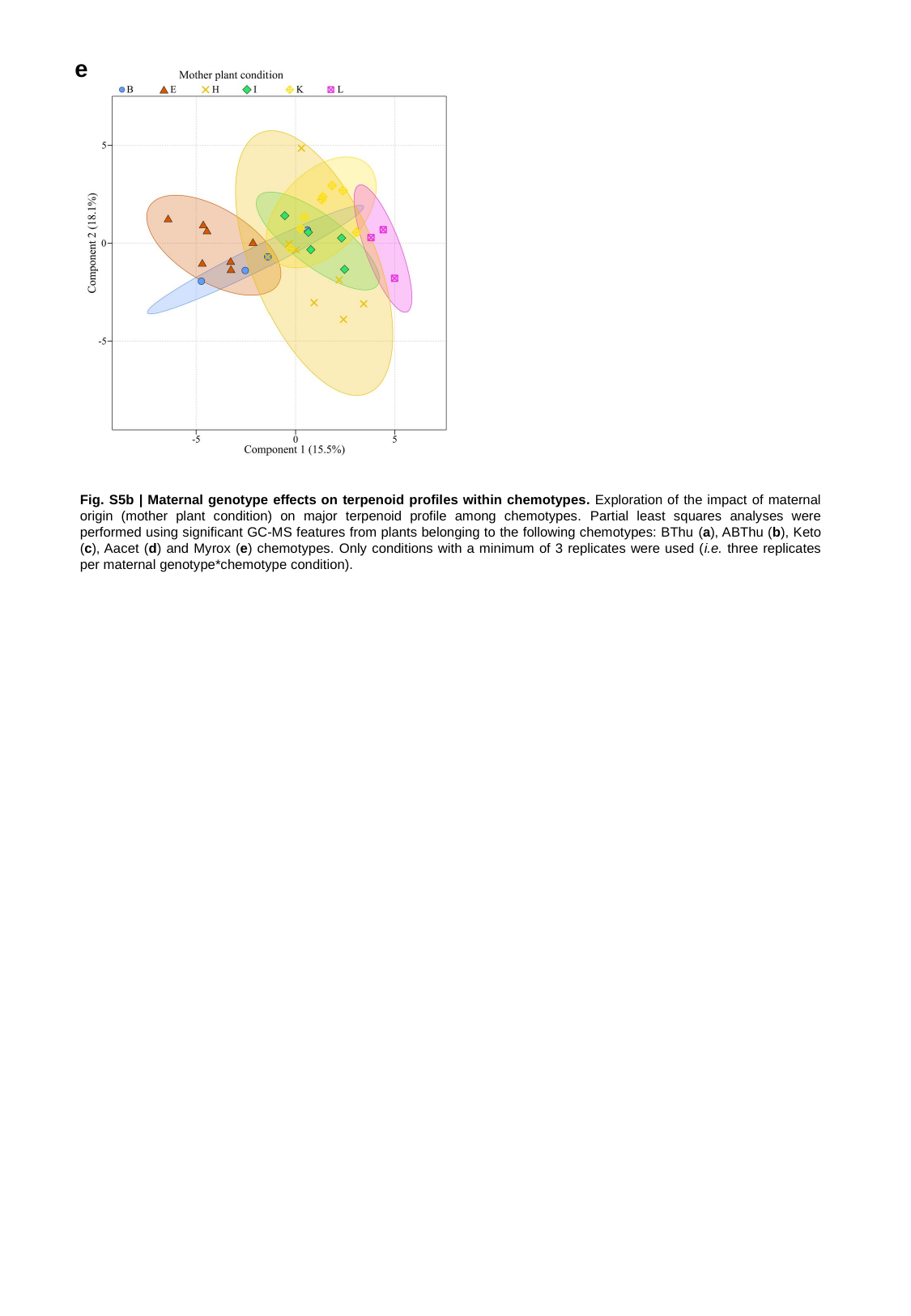

e
Fig. S5b | Maternal genotype effects on terpenoid profiles within chemotypes. Exploration of the impact of maternal origin (mother plant condition) on major terpenoid profile among chemotypes. Partial least squares analyses were performed using significant GC-MS features from plants belonging to the following chemotypes: BThu (a), ABThu (b), Keto (c), Aacet (d) and Myrox (e) chemotypes. Only conditions with a minimum of 3 replicates were used (i.e. three replicates per maternal genotype*chemotype condition).

## Slide 7
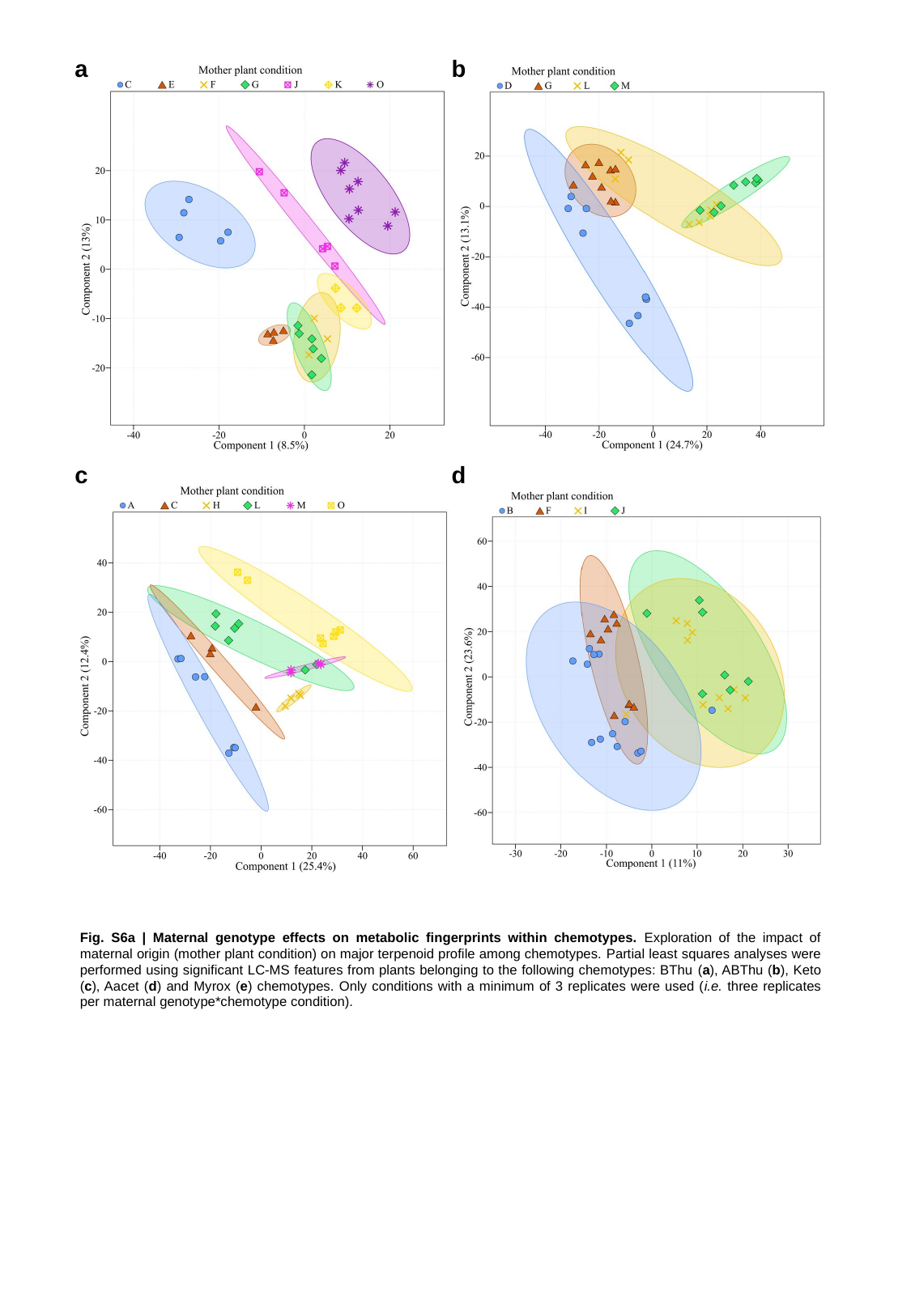

a
b
c
d
Fig. S6a | Maternal genotype effects on metabolic fingerprints within chemotypes. Exploration of the impact of maternal origin (mother plant condition) on major terpenoid profile among chemotypes. Partial least squares analyses were performed using significant LC-MS features from plants belonging to the following chemotypes: BThu (a), ABThu (b), Keto (c), Aacet (d) and Myrox (e) chemotypes. Only conditions with a minimum of 3 replicates were used (i.e. three replicates per maternal genotype*chemotype condition).

## Slide 8
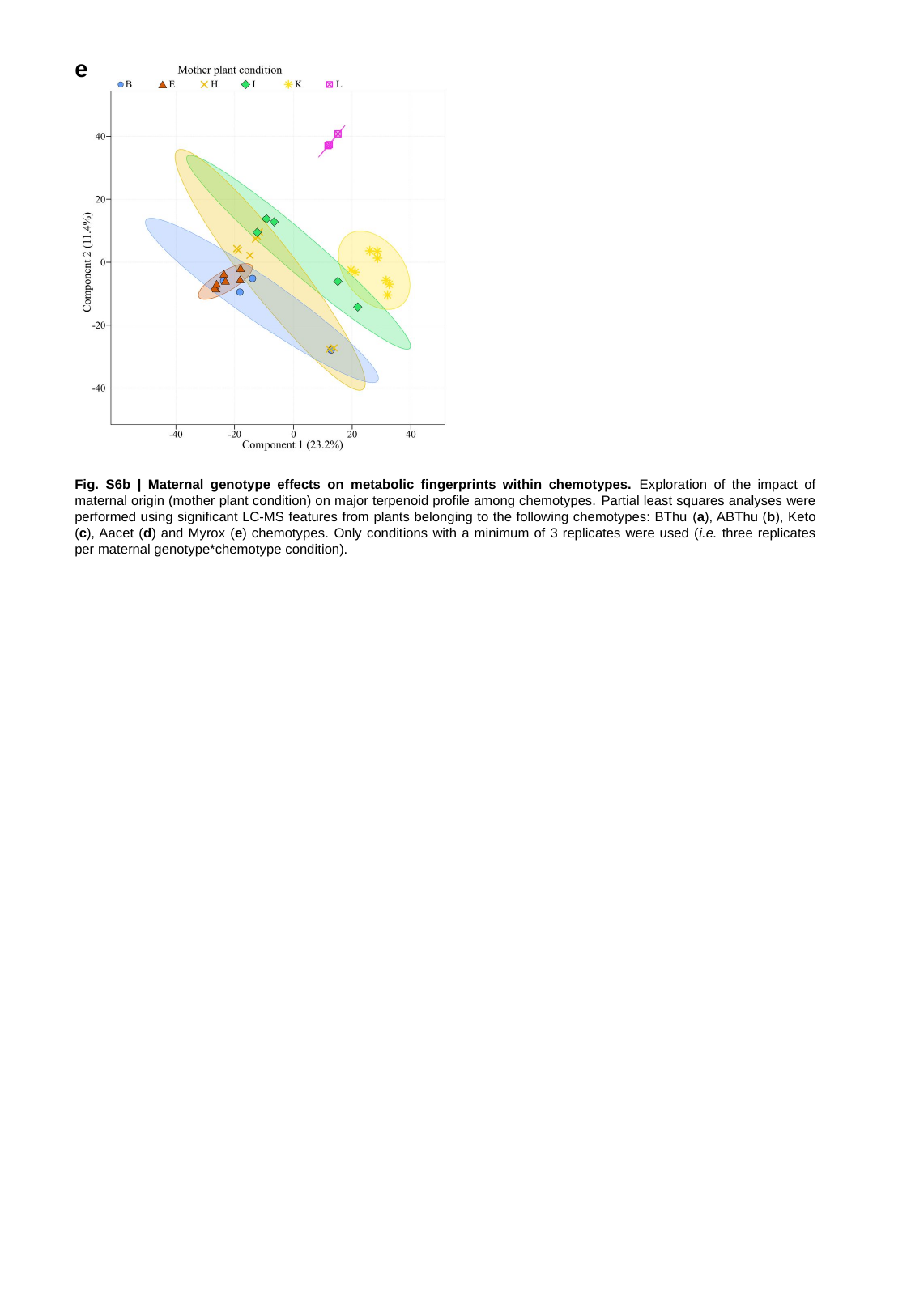

e
Fig. S6b | Maternal genotype effects on metabolic fingerprints within chemotypes. Exploration of the impact of maternal origin (mother plant condition) on major terpenoid profile among chemotypes. Partial least squares analyses were performed using significant LC-MS features from plants belonging to the following chemotypes: BThu (a), ABThu (b), Keto (c), Aacet (d) and Myrox (e) chemotypes. Only conditions with a minimum of 3 replicates were used (i.e. three replicates per maternal genotype*chemotype condition).
